# Supplementary material for: Modification of Microstructure and Mechanical Properties of Extruded AZ91-0.4Ce Magnesium Alloy through Addition of Ca
Source: Materials (Basel). 2024 Jul 8;17(13):3359. doi: 10.3390/ma17133359 (PMC11243162; doi:10.3390/ma17133359)
Supplement: Supplementary file 1 [file materials-17-03359-s001.zip › materials-3055230-supplementary.pdf]

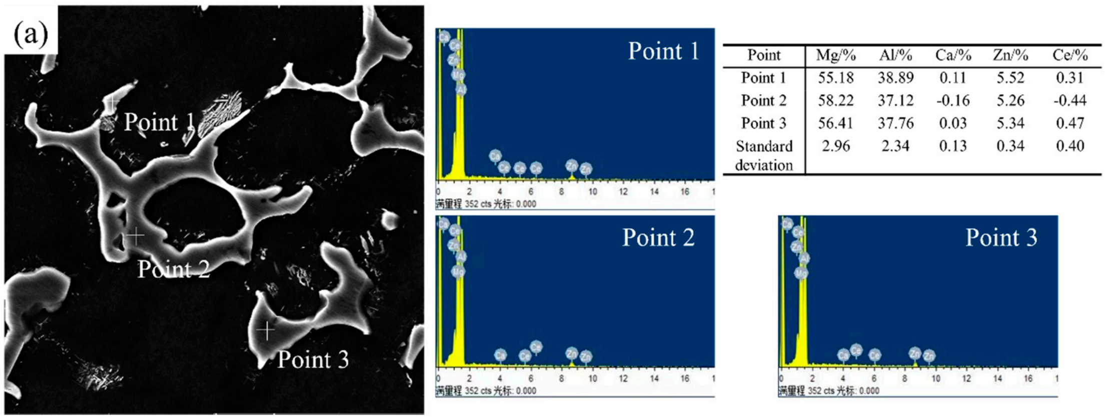

Point	Mg/%	Al/%	Ca/%	Zn/%	Ce/%
Point 1	55.18	38.89	0.11	5.52	0.31
Point 2	58.22	37.12	-0.16	5.26	-0.44
Point 3	56.41	37.76	0.03	5.34	0.47
Standard deviation	2.96	2.34	0.13	0.34	0.40

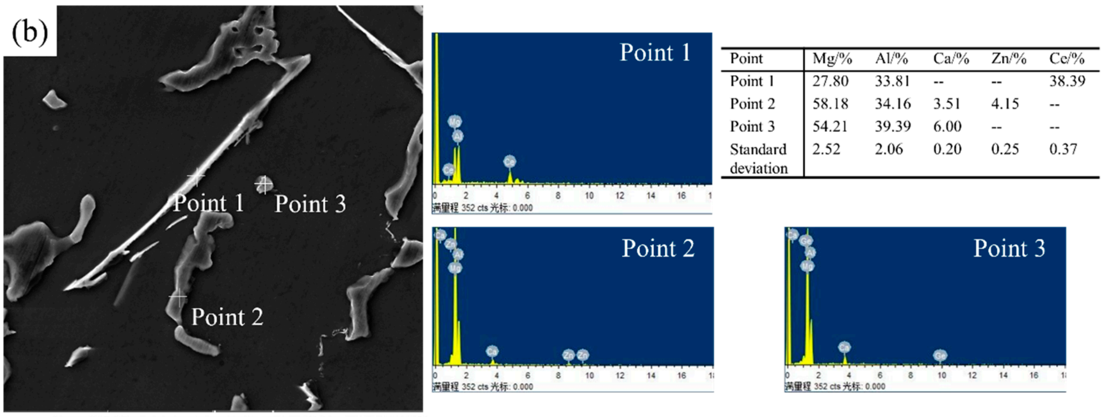

Point	Mg/%	Al/%	Ca/%	Zn/%	Ce/%
Point 1	27.80	33.81	--	--	38.39
Point 2	58.18	34.16	3.51	4.15	--
Point 3	54.21	39.39	6.00	--	--
Standard deviation	2.52	2.06	0.20	0.25	0.37

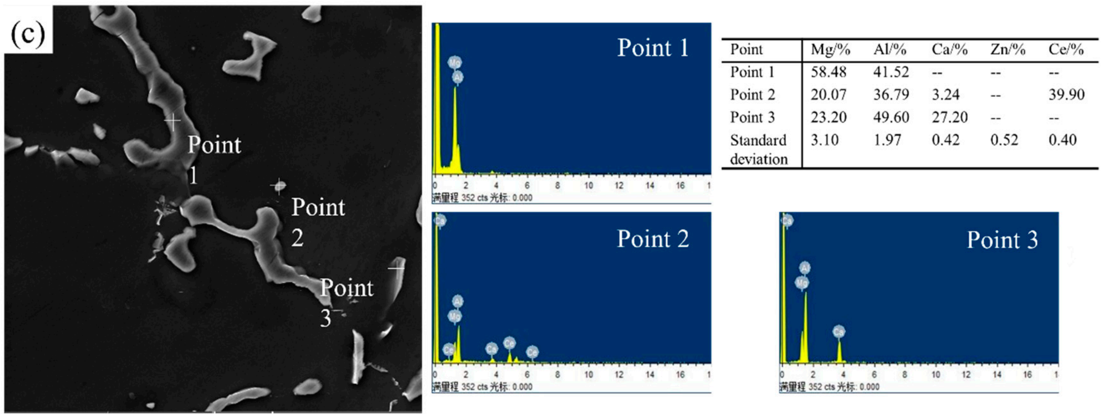

Point	Mg/%	Al/%	Ca/%	Zn/%	Ce/%
Point 1	58.48	41.52	--	--	--
Point 2	20.07	36.79	3.24	--	39.90
Point 3	23.20	49.60	27.20	--	--
Standard deviation	3.10	1.97	0.42	0.52	0.40

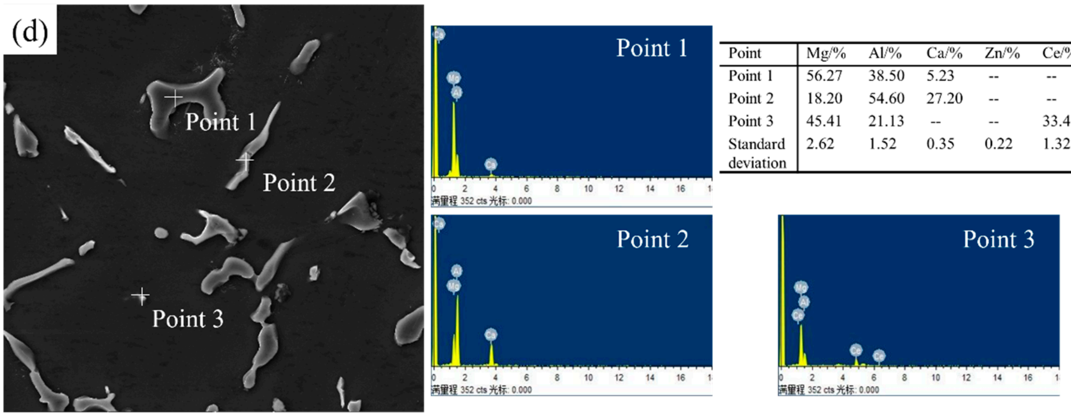

Point	Mg/%	Al/%	Ca/%	Zn/%	Ce/%
Point 1	56.27	38.50	5.23	--	--
Point 2	18.20	54.60	27.20	--	--
Point 3	45.41	21.13	--	--	33.46
Standard deviation	2.62	1.52	0.35	0.22	1.32

Figure S1 SEM image and EDS content of as-cast AZ91-0.4Ce-*x*Ca (*x*=0, 0.4, 0.8, 1.2wt.%) alloys. (a) AZ91-0.4Ce alloy, (b) AZ91-0.4Ce-0.4Ca alloy, (c) AZ91-0.4Ce-0.8Ca alloy, (d) AZ91-0.4Ce-1.2Ca alloy

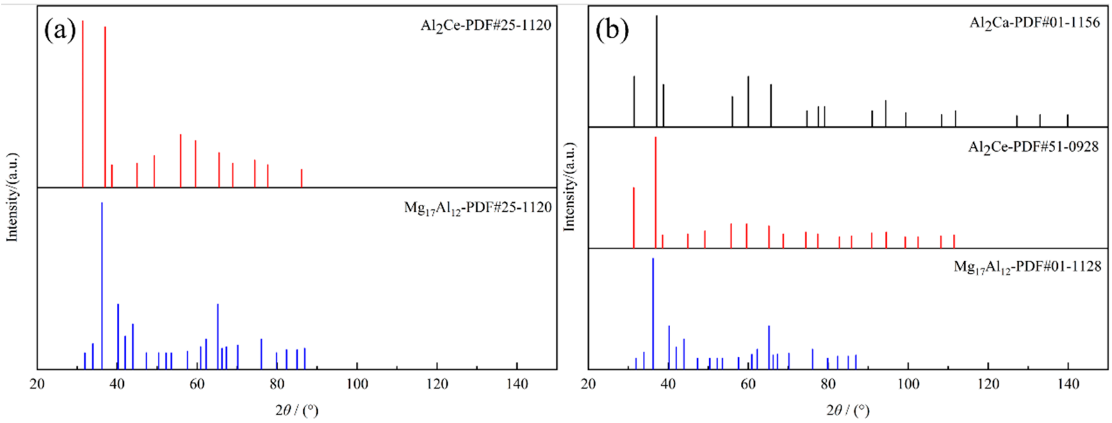

Figure S2 RD standard spectrum. (a)AZ91-0.4Ce; (b) AZ91-0.4Ce-xCa (x=0.4, 0.8, 1.2 wt.%)

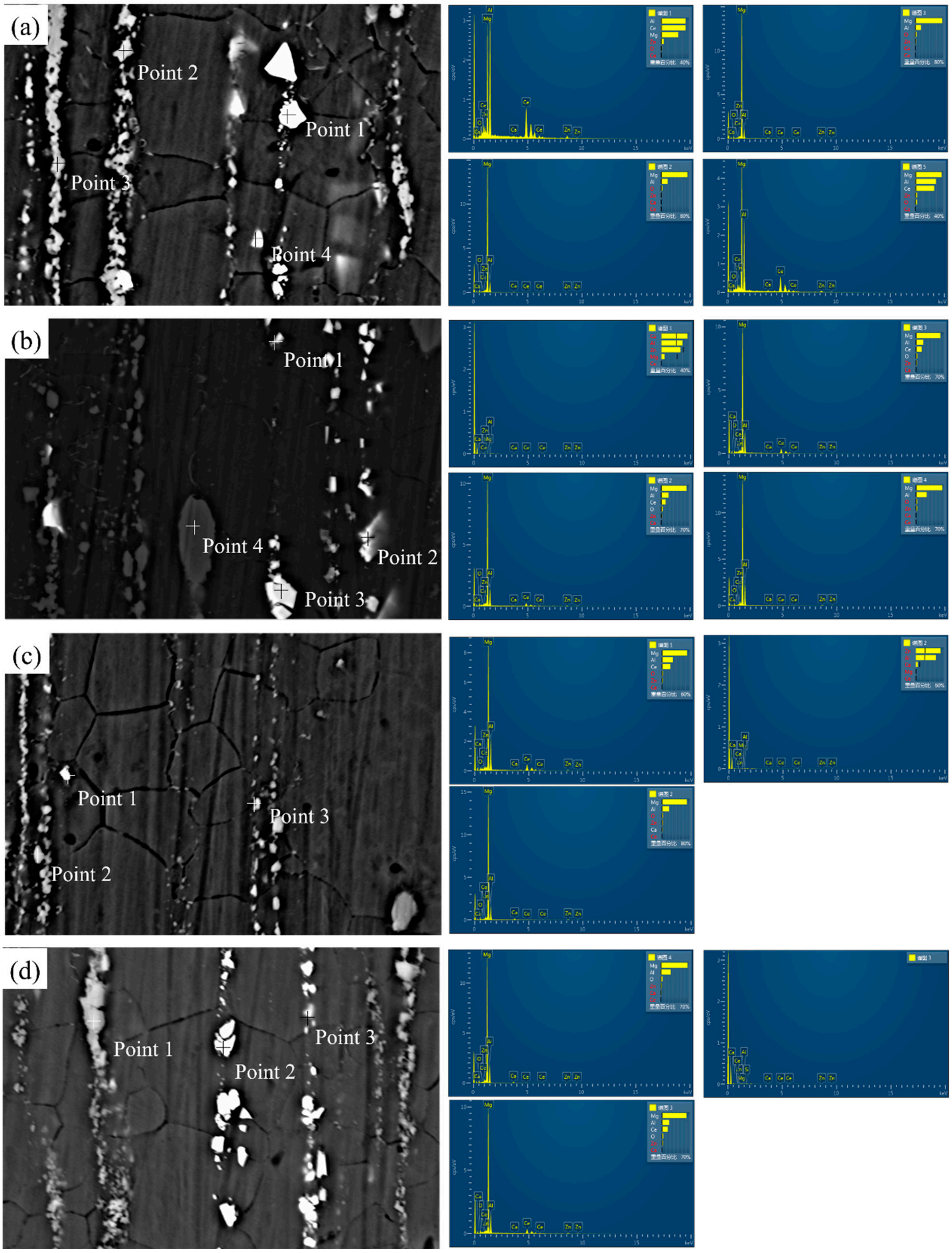

Figure S3 EM image and EDS content of extruded AZ91-0.4Ce-*x*Ca (*x*=0, 0.4, 0.8, 1.2wt.%) alloys. (a) AZ91-0.4Ce alloy, (b) AZ91-0.4Ce-0.4Ca alloy, (c) AZ91-0.4Ce-0.8Ca alloy, (d) AZ91-0.4Ce-1.2Ca alloy
